# Advancing psychological therapies for chronic pain

**DOI:** 10.12688/f1000research.10612.1

**Published:** 2017-04-11

**Authors:** Christopher Eccleston, Geert Crombez

**Affiliations:** 1 Centre for Pain Research, University of Bath, Bath, UK; 2 Department of Clinical-Experimental and Health Psychology, Ghent University, Gent, Belgium

**Keywords:** psychological therapies, chronic pain, non-pharmacological interventions, embodied pain

## Abstract

There is a strong tradition of therapy development and evaluation in the field of psychological interventions for chronic pain. However, despite this research production, the effects of treatments remain uncertain, and treatment development has stalled. This review summarises the current evidence but focusses on promising areas for improvement. Advancing psychological therapies for chronic pain will come from a radical re-imagining of the content, delivery, place, and control of therapy. The next generation of therapeutic interventions will also need alternative methods of measurement and evaluation, and options are discussed.

## Introduction

Psychological treatments—in particular, cognitive behaviour therapies—have been a mainstay of chronic pain management. The population of people who seek treatment for chronic pain is growing, and there is a rising incidence of chronic neuropathic pain ^1^, the growing realisation of the burden of pain in later life ^2^, and a recognition that performance of pharmacological interventions is disappointing ^3^. Despite the demand for treatment, progress in psychological therapy has now reached a turning point, and there is no clear direction on the route to take. This is a timely juncture to look critically at the evidence we have, to understand why treatment development is failing, and to consider how to cut a new path to clinical progress.

## Exploring the evidence base

The evidence for the efficacy of psychological interventions is largely underwhelming. There are four main Cochrane systematic reviews of psychological interventions for improving pain, affect, and disability in chronic pain—two with adults ^4, 5^ and two with adolescents ^4, 6^. This is not an under-researched area. Altogether, 101 randomised controlled trials (RCTs) have been conducted. For adults, behavioural and cognitive behavioural treatments show moderate-effect sizes of benefit over waiting lists and small or no effects over active comparators for outcomes in pain, disability, and mood. However, uncertainty over the effect estimates remains high because of poor-quality and small studies. For treatments of children and adolescents, there is moderate-quality evidence of efficacy of cognitive behavioural therapy (CBT), in particular for headache, and evidence is developing for musculoskeletal pain conditions such as fibromyalgia ^7^. The quality of recent trials in paediatrics is high, and there is innovation in methods of remote delivery ^8^. In paediatric pain, however, there is an historical absence of evidence for non-pain outcomes such as psychological and physical functioning, and for non-patient stakeholders such as parents or siblings. From 101 RCTs, the best conclusion we can draw is that there is low-quality evidence of small to moderate effects of CBT for chronic pain, meaning that the effect estimates could easily change with new evidence.

## Paradigm shift

Perhaps the next 101 trials will help us. Without change, however, we believe not. In 2013, we argued that there should be a halt on trial registration, until the quality and focus radically improve, because of a significant threat of research waste ^9^. There should be no new trials until three critical problems are addressed. First, treatment should be based on an extant model of behaviour change. In psychology, it is normal practice to run phase II or III studies without pre-clinical work or phase I study. *Post hoc* theorising is common. There should be a scientifically plausible reason for behaviour change, stated and mapped, and one should always assume the possibility of harm. Second, clinical endpoints of treatments and thresholds of treatment success should be established by the community. At present, the field is awash with therapist- or researcher-driven measurement. Outcomes developed and determined by patients, with meaningful endpoints, will help enormously. Dichotomous outcomes of meaningful changes in health state are rarely reported, relying instead on the use of continuous variables aggregated across groups. Third, innovation will come only by creating pathways from pre-clinical to clinical studies, by better understanding patient need, by resisting the errant individualisation of social problems which position responsibility for change with the individual alone, and by challenging the habit of pathologising normal, albeit maladaptive, behaviour. A new paradigm for developing innovative treatment is needed, requiring both theoretical and methodological attention.

## Novel targets for therapy development

There is promising work in four areas: in the search for common transdisciplinary mechanisms of therapeutic change, in better profiling of patient need and consequent tailoring of content, in exploring embodied pain models for analgesic as well as rehabilitative intervention, and in the use of computing technology to re-imagine therapeutic practice.

(1) Like surgery, psychological intervention is dependent on the skill, training, and experience of the operator; is manufactured in the moment; and is tailored to the individual case. The overall small- to moderate-effect sizes of CBT hide a heterogeneity of content, operator characteristics, exposure time, and therapist allegiance, which go largely unreported. Individual candidate measures of process are often investigated, but common features of treatment go undiscussed. Burns has argued recently that attention to common mechanisms, in particular ‘behavioural activation’—actively engaging in practicing or experimenting with meaningful physical change—is a good candidate ^10, 11^. A further example is the need to account for parental distress, and parenting, in the treatment of adolescent chronic pain ^12^. A novel target for therapy development is in making the non-specific specific.
(2) Despite a large evidence base, there are critical gaps. Individual differences and the importance of pain presentation are rarely investigated. There is some consideration of sex differences in therapy outcomes ^13^, of chronic pain in later life ^14^, and of delivery in particular settings such as the workplace ^15^. But these are rare cases. Odder still is the absence of data for specific populations. For example, we found only three RCTs of psychological interventions eligible for a Cochrane Review in chronic neuropathic pain ^16^. Exactly how the form and content of pain shape psychological experience is largely unexplored, hidden within large compound variables such as anxiety, depression, or disability. The extent to which, for example, worry about headache is critically different from worry about pelvic pain is important, as is the history of the meaning of that pain (for example, whether it was related to previous disease). A novel target for therapy development is establishing illness-specific psychological theory.
(3) Pain intrudes on awareness and functions to protect by urging escape and avoidance from potential harm. Repetitive inescapable interruption, motor preparation for flight, and heightened sensitivity to cues of danger create a pain-dominated environment in which accurate prediction of action in the context of pain is difficult ^17^. Recent theory in embodied pain suggests that the inherent uncertainty around experience might be a therapeutic opportunity, with potentially analgesic outcomes ^18^. Experience-altering interventions using virtual and augmented reality ^19^, anatomical education ^20^, or exposure ^21^ are interesting first forays into treatments aimed at directly altering experience. A fully embodied rehabilitation approach to chronic pain will embrace pain as always selected over competing demands for protection in the uncertain context of threat. A novel target for therapy development is to adopt an active psychology of meaningful engagement.
(4) A frontier for psychological therapy is to embrace the possibilities of technology, not only in augmenting, supporting, or replacing the remote delivery of traditional face-to-face treatment but for novel therapy content. Technology can do what therapists cannot, and can do many things better. Technology can accompany the patient, measure multiple aspects of experience, render data into accurate information instantaneously, give immediate access to knowledge, send and receive messages in near real-time, and allow discourse, anytime and almost anywhere. The opportunities of technology have yet to be explored. A user-centred modern therapy would be delivered flexibly over multiple devices, be highly dependent on the small data people trail about their lives ^22^, work just in time ^23^, and be highly contextual, integrated, and relational ^17^. A novel target for therapy development is to re-imagine therapy as active in the minutia of people’s lives, lives lived with technology rich in therapeutic promise.

## Development of methods

Next-generation therapies for chronic pain, indeed for psychological therapy in general, demand a new generation of methods. How we establish ‘what works for whom’ remains the critical challenge in pain science. Three areas deserve attention. First, there is a significant measurement problem in construct definition, independence, and relevance. Second, there is a unit-of-analysis problem. Individual experience is rarely investigated or reported, but the novel therapies discussed here will need sophisticated within-subject investigations. Finally, the quality of both conduct and reporting of studies needs to be considered and then improved.

(1) Psychology is largely concerned with behavioural assessment and change. Some behaviour is observable by self and other (for example, a physical attitude or gesture), and some is observable only by self (for example, a thought, emotion, belief, or bodily perception such as pain). The successful measurement of behaviour relies on the independence of a construct and on the quality of the measurement technology used to capture it. In pain, there is an active science of instrument development. Lost in pain research is a consideration of the importance of construct coherence even before the deployment of measurement technology. Here are three examples. We have shown that the measurement of acceptance of chronic pain is corrupted with content more pertinent to physical and social function, making it almost impossible to independently measure the role of function in altering acceptance, or vice versa ^24^. There is little evidence that adolescents experience catastrophic thinking about pain, and what is normally measured is better described as worry ^25^. And research into the popular construct of ‘somatisation’ is fundamentally flawed in pain research by the absence of demonstration that patients meet criteria for somatisation ^26^. Needed is consideration of the content of measures beyond their labels, the reporting of data at an item level, *a priori* establishment of independence of constructs, and a consensus over meaningful clinical endpoints.
(2) Also needed are within-subject idiographic methods. The randomised placebo-controlled trial is an ill-fitting method of assessing psychological treatment efficacy and safety and will be further challenged by individual, context-dependent, temporally dynamic therapies. Increased demands for data-sharing might allow for *post hoc* review of individual data, but they rely heavily on the availability and quality of trial data. Single-case series are often more relevant and are highly versatile and under-employed ^27^. In pain, there is also a strong tradition of using ecological momentary assessment in which people report on behaviour when it occurs or at set intervals of time ^28^. The advent of pervasive communication technology being repurposed for therapeutic use makes it necessary to innovate single-case, personally situated, data-rich methods. There is research already in the use of big data ^29^, and interesting development of a micro-randomised trial, that involves moving the randomisation point from pre-treatment to various points within treatment. For example, one might randomise the delivery of a remote message prompt, within-patient, within-trial ^23^.
(3) The conduct of all studies in pain is likely to come under further scrutiny for how known biases are managed and for the possibility of research misconduct, including fraud. Cochrane risk-of-bias assessment of all primary trials in psychological treatments shows general problems of reporting biases (for example, selective reporting) and performance bias (for example, lack of appropriate blinding). The emergence of reporting standards—for example, Consolidated Standards of Reporting Trials (CONSORT)—and the insistence by journals of trial registration are helpful developments; see www.equator-network.org. Further problems specific to trials of psychological interventions for chronic pain are the entering of patients to trial on the basis of criteria only indirectly relevant to treatment (for example, pain severity), entering of patients to trial with mild or no problems, failure to consider or report adverse events, and the selective or non-reporting of data. The extent to which these biases are systemic and arise from a failure of clinical equipoise in pain psychology is unknown, although there are examples of authors arguing for treatment efficacy, when their data show no evidence of effect ^30^. Understanding narrative bias will help authors judge the quality and impact of any efficacy investigation.

## Conclusions

There has been tremendous industry in producing a large number of RCTs, and even more uncontrolled evaluations, of psychological interventions for outcomes in chronic pain. But uncertainty over efficacy and harm remains. A radical re-imagining of therapy for chronic pain is needed, not least by a consideration of the role of technology improving access to existing therapy, and the therapy itself. Directly altering pain through a consideration of embodied perception—an *embodied pain* approach—offers exciting avenues for exploration ^18^. Making use of traces of data we leave from pervasive sensing and communication technology is especially promising. Methods of assessment and evaluation will also need to be developed, in particular to match therapy delivery that is tailored to individual needs or problems in ecological or natural environments. Guiding principles for advancing psychological therapies for chronic pain will be to ensure better translation from pre-clinical studies of pain and to protect equipoise from the threat of bias.

## References

[1] Fayaz A Croft P Langford RM: Prevalence of chronic pain in the UK: a systematic review and meta-analysis of population studies. *BMJ Open.* 2016;6(6):e010364. 10.1136/bmjopen-2015-010364 27324708 PMC4932255

[2] Reid MC Eccleston C Pillemer K: Management of chronic pain in older adults. *BMJ.* 2015;350:h532. 10.1136/bmj.h532 25680884 PMC4707527

[3] Moore A Derry S Eccleston C: Expect analgesic failure; pursue analgesic success. *BMJ.* 2013;346:f2690. 10.1136/bmj.f2690 23645858

[4] Eccleston C Fisher E Law E: Psychological interventions for parents of children and adolescents with chronic illness. *Cochrane Database Syst Rev.* 2015: (4): CD009660. 10.1002/14651858.CD009660.pub3 25874881 PMC4838404

[5] Eccleston C Fisher E Craig L: Psychological therapies (Internet-delivered) for the management of chronic pain in adults. *Cochrane Database Syst Rev.* 2014: (2): CD010152. 10.1002/14651858.CD010152.pub2 24574082 PMC6685592

[6] Fisher E Law E Palermo TM: Psychological therapies (remotely delivered) for the management of chronic and recurrent pain in children and adolescents. *Cochrane Database Syst Rev.* 2015: (3): CD011118. 10.1002/14651858.CD011118.pub2 25803793 PMC4833498

[7] Kashikar-Zuck S Ting TV Arnold LM: Cognitive behavioral therapy for the treatment of juvenile fibromyalgia: a multisite, single-blind, randomized, controlled clinical trial. *Arthritis Rheum.* 2012;64(1):297–305. 10.1002/art.30644 22108765 PMC3690664

[8] Palermo TM Law EF Fales J: Internet-delivered cognitive-behavioral treatment for adolescents with chronic pain and their parents: a randomized controlled multicenter trial. *Pain.* 2016;157(1):174–85. 10.1097/j.pain.0000000000000348 26335910 PMC4852469

[9] Morley S Williams A Eccleston C: Examining the evidence about psychological treatments for chronic pain: time for a paradigm shift? *Pain.* 2013;154(10):1929–31. 10.1016/j.pain.2013.05.049 23742793

[10] Burns JW: Mechanisms, mechanisms, mechanisms: it really does all boil down to mechanisms. *Pain.* 2016;157(11):2393–4. 10.1097/j.pain.0000000000000696 27548048 PMC5846486

[11] Burns JW Nielson WR Jensen MP: Specific and general therapeutic mechanisms in cognitive behavioral treatment of chronic pain. *J Consult Clin Psychol.* 2015;83(1):1–11. 10.1037/a0037208 24979313

[12] Palermo TM Law EF Essner B: Adaptation of Problem-Solving Skills Training (PSST) for Parent Caregivers of Youth with Chronic Pain. *Clin Pract Pediatr Psychol.* 2014;2(3):212–23. 10.1037/cpp0000067 25422795 PMC4239695

[13] Boerner KE Eccleston C Chambers CT: Sex differences in the efficacy of psychological therapies for the management of chronic and recurrent pain in children and adolescents: A systematic review and meta-analysis. *Pain.* 2016. 10.1097/j.pain.0000000000000803 27984491

[14] Keefe FJ Porter L Somers T: Psychosocial interventions for managing pain in older adults: outcomes and clinical implications. *Br J Anaesth.* 2013;111(1):89–94. 10.1093/bja/aet129 23794650 PMC3690316

[15] Linton SJ Boersma K Traczyk M: Early Workplace Communication and Problem Solving to Prevent Back Disability: Results of a Randomized Controlled Trial Among High-Risk Workers and Their Supervisors. *J Occup Rehabil.* 2016;26(2):150–9. 10.1007/s10926-015-9596-z 26202039 PMC4854941

[16] Eccleston C Hearn L Williams AC: Psychological therapies for the management of chronic neuropathic pain in adults. *Cochrane Database Syst Rev.* 2015; (10): CD011259. 10.1002/14651858.CD011259.pub2 26513427 PMC6485637

[17] Crombez G Eccleston C Van Damme S: Fear-avoidance model of chronic pain: the next generation. *Clin J Pain.* 2012;28(6):475–83. 10.1097/AJP.0b013e3182385392 22673479

[18] Tabor A Keogh E Eccleston C: Embodied pain-negotiating the boundaries of possible action. *Pain.* 2017. 10.1097/j.pain.0000000000000875 28195855

[19] Penelle B Mouraux D Brassinne E: 3D augmented reality applied to the treatment of neuropathic pain. *Proc 9th Intl Conf Disability, Virtual Reality & Associated Technologies.*In PM Sharkey & E Klinger (Eds), Laval, France.2012;61–68. Reference Source

[20] Louw A Diener I Landers MR: Preoperative pain neuroscience education for lumbar radiculopathy: a multicenter randomized controlled trial with 1-year follow-up. *Spine (Phila Pa 1976).* 2014;39(18):1449–57. 10.1097/BRS.0000000000000444 24875964

[21] den Hollander M Goossens M de Jong J: Expose or protect? A randomized controlled trial of exposure in vivo vs pain-contingent treatment as usual in patients with complex regional pain syndrome type 1. *Pain.* 2016;157(10):2318–29. 10.1097/j.pain.0000000000000651 27429174

[22] Estrin D: Small data, where n = me. *Communications of the ACM.* 2014;57(4):32–34. 10.1145/2580944

[23] Klasnja P Hekler EB Shiffman S: Microrandomized trials: An experimental design for developing just-in-time adaptive interventions. *Health Psychol.* 2015;34S:1220–8. 10.1037/hea0000305 26651463 PMC4732571

[24] Lauwerier E Caes L Van Damme S: Acceptance: what's in a name? A content analysis of acceptance instruments in individuals with chronic pain. *J Pain.* 2015;16(4):306–17. 10.1016/j.jpain.2015.01.001 25584430

[25] Eccleston C Fisher EA Vervoort T: Worry and catastrophizing about pain in youth: a reappraisal. *Pain.* 2012;153(8):1560–2. 10.1016/j.pain.2012.02.039 22516589

[26] Crombez G Beirens K Van Damme S: The unbearable lightness of somatisation: a systematic review of the concept of somatisation in empirical studies of pain. *Pain.* 2009;145(1–2):31–5. 10.1016/j.pain.2009.04.006 19427734

[27] Onghena P Edgington ES: Customization of pain treatments: single-case design and analysis. *Clin J Pain.* 2005;21(1):56–68; discussion69–72. 10.1097/00002508-200501000-00007 15599132

[28] Garcia-Palacios A Herrero R Belmonte MA: Ecological momentary assessment for chronic pain in fibromyalgia using a smartphone: a randomized crossover study. *Eur J Pain.* 2014;18(6):862–72. 10.1002/j.1532-2149.2013.00425.x 24921074

[29] Lee H McAuley JH Hübscher M: Tweeting back: predicting new cases of back pain with mass social media data. *J Am Med Inform Assoc.* 2016;23(3):644–8. 10.1093/jamia/ocv168 26661720 PMC7839924

[30] Wetherell JL Afari N Rutledge T: A randomized, controlled trial of acceptance and commitment therapy and cognitive-behavioral therapy for chronic pain. *Pain.* 2011;152(9):2098–107. 10.1016/j.pain.2011.05.016 21683527

